# The impact of COVID-19 pandemic on anxiety and depression among physical therapists in Saudi Arabia: a cross-sectional study

**DOI:** 10.1186/s12909-022-03785-x

**Published:** 2022-11-01

**Authors:** Abeer Hamza Abdulghani, Tauseef Ahmad, Hamza Mohammad Abdulghani

**Affiliations:** 1 College of Applied Medical Sciences, King Saud University, Riyadh, Saudi Arabia; 2 College of Medicine, King Saud University, Riyadh, Saudi Arabia; 3 Department of Medical Education, Head of The Assessment & Evaluation Centre, College of Medicine, King Saud University, Riyadh, 11321 Saudi Arabia

**Keywords:** Anxiety, Depression, Physical therapists, Mental health, COVID19

## Abstract

**Backgrounds:**

A physical therapist may become infected while treating a patient since they are in direct contact with them or within a two-meter radius. In addition, physical therapists may feel that they are more susceptible to COVID-19 infection when applying rehabilitation practices, which often involve direct contact with patients. The physical therapist were surveyed on their level of anxiety and depression due to the Coronavirus disease pandemic (COVID-19).

**Methods:**

The physical therapists were asked to complete two reliable and validated scales, the Generalized Anxiety Disorder scale (GAD-7) and the Patient Health Questionnaire-9 (PHQ-9), to identify the presence of anxiety and depression in the participants. In addition, logistic regression models were used to determine the general characteristics of anxiety or depression.

**Results:**

Among the 117 physical therapists who completed and participated in the study, 74 (63%) and 65 (55.5%) physical therapists reported having symptoms of anxiety and depression, respectively. The prevalence of overall anxiety levels was higher; mild (OR = 2.09; *P* = 0.08), moderate (OR = 2.26; *P* = 0.15), and severe levels six times as high (OR = 6.28; *P* = 0.1) in females compared to male physical therapists. Females, younger age, unmarried individuals, not having children, and not living with family showed a higher prevalence of anxiety and depression. Binary logistic regression analysis also revealed that the female gender, a single individual, and having no children were associated with anxiety and depression.

**Conclusions:**

A significant percentage of physical therapists reported symptoms of anxiety and depression, especially among females, younger age, single individuals, not having children, and not living with family. Thus, the mental health of physical therapists is suggested to be constantly and cautiously monitored, especially for those at high risk of developing psychological symptoms.

## Introduction

Coronavirus disease (COVID-19) is an infectious disease caused by the severe acute respiratory syndrome coronavirus 2 (SARS-CoV-2). It first emerged in December 2019 in the city of Wuhan, China. The virus has been spreading domestically and globally ever since, affecting nearly all countries worldwide [1]. Since then, COVID-19 has been described as an international public health issue that has overwhelmed many health systems globally. On March 2, the first case that tested positive for COVID-19 was reported by the Ministry of Health in Saudi Arabia [2].

The impact of COVID-19 on the general population's mental health has been well researched since the pandemic’s beginning. In addition to the fear of infection and the preventive measures imposed, including social distancing, isolation, quarantine, and job loss, the general population has been exposed to stressors [3]. Stressors of this kind can impair mental health, particularly among vulnerable populations like the elderly, chronically ill individuals, and low-income families [4]. People who work in hospitals are more exposed to infectious diseases than the general population due to their vital and massive presence during the pandemic as front liners [5]. A prospective population-based study investigating the risk of testing positive for COVID-19 among frontline healthcare workers (HCWs) compared with the larger community in the UK and the USA, showed a higher risk of infection among frontline HCWs [6].

Healthcare providers are not only at the risk of death from infection but are more vulnerable to anxiety and stress due to overwhelming health care systems, in addition to the fear of acquiring a virus infection [6, 7]. Several lines of the research reported an increase in psychological distress during the COVID-19 pandemic among the general population and HCWs [8–10]. Thus, there is an urgent call for more action and attention to address the impact on HCWs and assist people through this challenging time. A local study on HCWs at the Ministry of National Guard-Health Affairs reported a significantly higher mean on the level of concern among HCWs about the COVID-19 pandemic, with 27.9% of HCWs having high concern, 46.9% moderate concern, and 25.2% low concern [11].

WHO has defined "contact with COVID-19 as close direct contact with any individual within six feet of an infected person for a total of 15 min or more" [12]. The virus that causes COVID-19 spreads mainly through person-to-person transmission through contact or droplet transmission [13]. As a result, it may hazard the health of HCWs, including physical therapists, who risk of exposure due to the nature of their occupation. In addition, physical therapists may feel that they are more prone to COVID-19 infection during the application of rehabilitation practice, which often involves direct contact with patients.

A study investigated the mental health burden of COVID-19 on Korean physical therapists; results indicated that one-third of them had anxiety, and another one-third had depression. Among them, 12.5% were in their 40 s, and 37.5% were in their 50 s. In addition the results reported that physical therapists in their 30 s and 50 s had a significantly higher risk of depression than those who were less than 30 years of age [1].

A recently published study evaluated stress perception and identified associated factors among Brazilian physical therapists during the COVID-19 pandemic. The results showed an increased stress level associated with several factors, including female workers, younger age, previous diagnosis of depressive or anxiety disorder, and worsening in sleep quality. Also, a large reduction in family income, housework, relationship with the partner, concern about close people/family members being infected with SARS-CoV-2, and loneliness were associated with stress [14]. Different countries have different pandemic situations, and some have a higher death rate than others. Following WHO Health Emergency Dashboard, the USA had 94,237,260 confirmed cases of COVID-19 and 1,041,323 deaths; the UK had 23,585,309 confirmed cases of COVID-19 with 189,484 deaths; the UAE had 1,021,191 confirmed cases of COVID-19 with 2342 deaths. Similarly, some countries have a high number of COVID-19 cases, while others have fewer cases of COVID-19. Saudi Arabia is also affected by the COVID pandemic, with 814,829 reported cases and 9,322 deaths [2, 15]. While reviewing these studies, other confounders could be considered these confounders may play an important role in psychological distress, including the presence of prior psychiatric history, coping styles, culture, support systems, and infection of family members [16]. The rise in stress levels among health care workers contributes to work burnout and may lead to decreased patient care quality and practice safety. The result has been an increased risk of patient safety, poor quality of treatment, and decreased patient satisfaction. The uncertainties and fears associated with the virus outbreak, mass lockdowns, and economic recession, are predicted to lead to increases in mental disorders, including suicidal thoughts or attempts [17]. Different countries faced different pandemic situations. The death rate in some countries is higher than in others. Similarly, some countries have an increase number of COVID-19 cases, while others have fewer cases of COVID.

An extensive literature review showed no local study had investigated this important topic among physical therapists in Saudi Arabia. Therefore, this study aimed to investigate the mental health burden of the COVID-19 pandemic on the level of anxiety and depression among physical therapists in Saudi Arabia. Also, it evaluated the association of sociodemographic factors with the risk of anxiety and depression.

## Materials and methods

### Design and participants

This cross-sectional study was conducted in February 2021. The participants selected in this study were physical therapists working at private or governmental hospitals and living in Saudi Arabia. Inclusion criteria were Saudi physical therapists who had worked in person with patients during the COVID-19 pandemic with at least one year of experience. In addition, the exclusion criteria were students, interns, previous diagnosis of a psychiatric illness, and history of confirmed infection with COVID-19 in the selected participants. Invitation to the study was sent electronically to the participants through an e-mail and social media platforms; it contained a link to the online survey.

### Outcome measure

A self-administered online survey was sent through e-mail, and the link was shared on social media. The questionnaire comprised two parts: the first part consisted of participants' sociodemographic data (age, sex, marital status, number of children, family members living together, previous medical history), clinical and work characteristics, and information related to COVID-19 (clinical practice setting, years of clinical experience, exposure to known positive tested COVID-19 patients, isolation experience due to contact with COVID-19 patients, and the presence of family members infected with COVID-19). The second part was screening for the existence of anxiety and depression using the Generalized Anxiety Disorder scale (GAD-7) (Appendix 1) and the Patient Health Questionnaire-9 (PHQ-9) (Appendix 2). These two scales are reliable, validated, and well-known measures of depression and anxiety symptoms for patients’ care within a primary or secondary mental health service [18].

GAD-7 is a self-report scale consisting of a seven-item anxiety scale for generalized anxiety symptoms. Total scores range from 0 to 21 and are classified as 'mild' (5–9), 'moderate' (10–14), and 'severe' (15–21). A cut-off value of ≥ 10 was identified that optimized sensitivity (89%) and specificity (82%) [19]. In addition the participants reported how often they had been bothered by generalized anxiety symptoms over the past two weeks (e.g., feeling nervous, unable to stop or control worrying, becoming easily annoyed or irritable). The reported alpha reliability for GAD-7 was 0.80 [20].

PHQ-9 is a nine-item patient self-report depression scale with total scores ranging from 0 to 27 (cut-off value: ≥ 10). A cut-off score was optimized: sensitivity 0.88, 95% confidence interval 0.83 to 0.92; specificity 0.85, 0.82 to 0.88 [21]. Participants reported how often they had been bothered by depression symptoms over the last two weeks (e.g., feeling hopeless, lack of interest or pleasure in doing things, negative self-evaluation). The response options of "not at all = 0", "several days = 1", "more than half the days = 2", and "nearly every day = 3". Scores are classified as 'mild' (5–9), 'moderate' (10–14), 'moderately severe' (15–19), and 'severe' (20–27). The reported alpha reliability for PHQ-9 was 0.85 [22].

### Statistical analysis

Statistical analysis of the study was performed using Statistical Package for the Social Sciences (SPSS) version 25.0 software. All descriptive analyses were performed using mean, standard deviation, percentage, and frequency. In addition, Chi-square and t-tests were conducted to analyze the presence of anxiety and depression relative to the sociodemographic characteristics. Also, binary logistic regression analysis was conducted for results regarding risk factors. The acceptable statistical significance was set at *p* < 0.05. Data in the current results show approximately normal distribution.

### Sample size

Sample size = Z _1-a/2_^2^ (SD) ^2^ / d.^2^

Z_1-a/2_^2^ = Is standard normal variate (at 5% type 1 error (*p* < 0.05) it is 1.96 and 1% type 1 error (*p* > 0.01) it is 2.58). In our case, *P* values are considered significant below 0.05; hence 1.96 is used in the formula.

d = Absolute error 5%

SD = Expected proportion in population proportion based on current studies, previous studies or pilot studies. (Many previously published studies during COVID-19 actual number of physical therapist’s sample not more than 27%, some studies reported 27%, 24%, and some 11%).

Sample size = 1.96^2^ * (27)^2^ / 5^2^ = 111.97.

Sample size if 27% according to previous published studies = 112.

The minimum requirement for conducting any study sample size must be 112 or more (based on previously published studies).

### Participants

A total of 131 physical therapists agreed to take part in the study. After a careful evaluation, 117 physical therapists completed responses and were included in the study, with a response rate of 89.3%. Personal and sociodemographic information of the participants was presented in Table 1. Among 117 physical therapists, there were 45 (38.5%) males and 72 (61.5%) females. The mean age of the participants was 25.6 ± 1.3 (mean ± SD). An almost equal number of participants in the current study were single, 55 (47.0%) and married, 56 (47.9%).

**Table 1:** Demographic data of physical therapists

Item	Categories	N(%)
Gender	Male	45(38.5)
	Female	72(61.5)
Age	≤ 25 year	21(17.9)
	26–35 year	74(63.2)
	36–45 year	18(15.4)
	45–55 year	3(2.6)
	56–65 year	1(0.9)
Marital Status	Single	55(47.0)
	Married	56(47.9)
	Divorced	6(5.1)
Do you have children	Yes	39(33.3)
	No	78(66.7)
You live with your family	Yes	95(81.2)
	No	22(18.8)
Do you have a child between the age (0 to 6)?	Yes	33(28.2)
	No	84(71.8)
Do you live with a family member aged ≥ 65?	Yes	43(36.8)
	No	74(63.2)
Medical problem	Yes	16(13.7)
	No	101(86.3)
Current working region	Central region	68(58.1)
	East region	11(9.4)
	West region	24(20.5)
	South region	7(6.0)
	North region	7(6.0)
what is your current practice setting?	General hospital	81(69.2)
	Private clinic	28(23.9)
	Others	8(6.8)
Years of clinical experience	< 2 year	24(20.5)
	2 to 5 year	47(40.2)
	5–10 year	22(18.8)
	≥ 10 year	24(20.5)
Isolation experience	Yes	37(31.6)
	No	80(68.4)
Had any of your family members been infected with COVID-19	Yes	51(43.6)
	No	66(56.4)
Working in-person care during the pandemic?	Yes	80(68.4)
	No	37(31.6)
Taking care of COVID-19 patients	Yes	26(22.2)
	No	91(77.8)
Anxiety level	No anxiety	43(36.8)
	Mild Anxiety	48(41.0)
	Moderate anxiety	19(16.2)
	Severe anxiety	7(6.0)
Depression Level	No	52(44.4)
	Mild	38(32.5)
	Moderate	21(17.9)
	Severe	6(5.1)

## Results

Most of the physical therapists, 78 (66.7%), had children. Moreover, 95 (81.2%) of participants lived with their families. About 101 (86.3%) of participants had no medical problems, and most of them, 81 (69.2%), worked in a governmental hospital. Furthermore, 37 (31.6%) of physical therapists had isolation experiences due to contact with positive COVID-19 patients, and 51 (43.6%) participants reported that their family member was infected with COVID-19. Among the participants, 22.2% of physical therapists cared for COVID-19 patients (Table 1).

Based on the GAD-7 scale, the levels of anxiety were reported as no anxiety 43 (36.8%), mild anxiety 48 (41.0%), moderate anxiety 19 (16.2%), and severe anxiety 7 (6.0%). Similarly, based on the PHQ-9 scale, the levels of depression were reported as no depression 52 (44.4%), mild depression 38(32.5%), moderate depression 21 (17.9%), and severe depression 6 (5.1%) (Table 1). In addition the majority of the participants had good work experience, among them less than two years, 24 (20.5%), 2 to 5 years 47 (40.2%), 5 to 10 years 22 (18.8%), and more than ten years 24 (20.5%) (Table 1).

In the current study, physical therapists' anxiety levels were associated with different variables. According to the participant's age, the younger individuals (26–35 years) had more anxiety levels (severe anxiety for less than 25 years was 42.9%, and severe anxiety for 26–35 years was 57.1%) compared to other age groups. Similarly, those who were single had more anxiety (moderate-57.9%, and severe-57.1) than the married participants. Those with no children had more anxiety as mild-62.5%, moderate-73.7%, and severe-85.7%. Furthermore, those physical therapists taking care of COVID-19 patients had lower anxiety levels than those were not attending COVID-19 patients 26 (22.2%) and severe 7 (100%). However, no factors showed a statistically significant difference between physical therapists with anxiety and those without anxiety (*P* > 0.05) (Table 2).

**Table 2:** Associations between Anxiety levels and different variables

			**Anxiety**				
**Item**	**Categories**	**N(%)**	**No (43)**	**Mild (48)**	**Moderate (19)**	**Severe (7)**	**χ2 *(P*-value)**
Gender	Male	45(38.5)	22(51.2)	16(33.3)	6(31.6)	1(14.3)	5.57(0.13)
	Female	72(61.5)	21(48.8)	32(66.7)	13(68.4)	6(85.7)	
Age	≤ 25 year	21(17.9)	7(16.3)	7(14.6)	4(21.1)	3(42.9)	8.89(0.71)
	26–35 year	74(63.2)	28(65.1)	32(66.7)	10(52.6)	4(57.1)	
	36–45 year	18(15.4)	6(14.0)	7(14.6)	5(26.3)	0(0.0)	
	45–55 year	3(2.6)	1(2.3)	2(4.2)	0(0.0)	0(0.0)	
	56–65 year	1(0.9)	1(2.3)	0(0.0)	0(0.0)	0(0.0)	
Marital Status	Single	55(47.0)	20(46.5)	20(41.7)	11(57.9)	4(57.1)	3.56(0.73)
	Married	56(47.9)	22(51.2)	24(50.0)	7(36.8)	3(42.9)	
	Divorced	6(5.1)	1(2.3)	4(8.3)	1(5.3)	0(0.0)	
Do you have children	Yes	39(33.3)	15(34.9)	18(37.5)	5(26.3)	1(14.3)	1.98(0.57)
	No	78(66.7)	28(65.1)	30(62.5)	14(73.7)	6(85.7)	
You live with your family	Yes	95(81.2)	38(88.4)	38(79.2)	14(73.7)	5(71.4)	2.71(0.43)
	No	22(18.8)	5(11.6)	10(20.8)	5(26.3)	2(28.6)	
Do you have a child between the age (0 to 6)	Yes	33(28.2)	12(27.9)	15(31.3)	5(26.3)	1(14.3)	0.92(0.81)
	No	84(71.8)	31(72.1)	33(68.8)	14(73.7)	6(85.7)	
Do you live with a family member aged ≥ 65	Yes	43(36.8)	16(37.2)	18(37.5)	7(36.8)	2(28.6)	0.21(0.97)
	No	74(63.2)	27(62.8)	30(62.5)	12(63.2)	5(71.4)	
Medical problem	Yes	16(13.7)	7(16.3)	5(10.4)	2(10.5)	2(28.6)	2.15(0.54)
	No	101(86.3)	36(83.7)	43(89.6)	17(89.5)	5(71.4)	
Current working region	Central region	68(58.1)	25(58.1)	27(56.3)	12(63.2)	4(57.1)	5.06(0.95)
	East region	11(9.4)	3(7.0)	5(10.4)	2(10.5)	1(14.3)	
	West region	24(20.5)	10(23.3)	10(20.8)	3(15.8)	1(14.3)	
	South region	7(6.0)	3(7.0)	2(4.2)	2(10.5)	0(0.0)	
	North region	7(6.0)	2(4.7)	4(8.3)	0(0.0)	1(14.3)	
what is your current practice setting	General hospital	81(69.2)	29(67.4)	35(72.9)	14(73.7)	3(42.9)	14.29(0.02)
	Private clinic	28(23.9)	8(18.6)	13(27.1)	5(26.3)	2(28.6)	
	Others	8(6.8)	6(14.2)	0(0.0)	0(0.0)	2(28.6)	
Years of clinical experience	< 2 year	24(20.5)	8(18.6)	8(16.7)	5(26.3)	3(42.9)	8.70(0.46)
	2 to 5 year	47(40.2)	15(34.9)	24(50.0)	6(31.6)	2(28.6)	
	5–10 year	22(18.8)	9(20.9)	6(12.5)	5(26.3)	2(28.6)	
	≥ 10 year	24(20.5)	11(25.6)	10(20.8)	3(15.8)	0(0.0)	
Isolation experience	Yes	37(31.6)	9(20.9)	18(37.5)	7(36.8)	3(42.9)	3.68(0.29)
	No	80(68.4)	34(79.1)	30(62.5)	12(63.2)	4(57.1)	
Had any of your family members been infected with COVID-19	Yes	51(43.6)	22(51.2)	17(35.4)	10(52.6)	2(28.6)	3.58(0.31)
	No	66(56.4)	21(48.8)	31(64.6)	9(47.4)	5(71.4)	
working in-person care (in clinic) as a physical therapist during the pandemic?	Yes	80(68.4)	27(62.8)	34(70.8)	14(73.7)	5(71.4)	1.03(0.79)
	No	37(31.6)	16(37.2)	14(29.2)	5(26.3)	2(28.6)	
Taking care of COVID-19 patients	Yes	26(22.2)	11(25.6)	11(22.9)	4(21.1)	0(0.0)	2.30(0.51)
	No	91(77.8)	32(74.4)	37(77.1)	15(78.9)	7(100)	

Table 3 summarizes the association of different variables with depression during the COVID-19 pandemic. Most female physical therapists had depression levels significantly higher (*P* = 0.02) compared to males. Similar to the anxiety findings, Similar to the anxiety findings, the younger physical therapists had more depression as compared to other age groups. Those physical therapists with no children had significantly more depression (*P* = 0.01). There was also a significant finding in the current study that physical therapists who had a child aged six years or younger, had less depression (*P* = 0.05). In addition, those physical therapists living with a family member aged 65 years or older experienced less depression (*P* = 0.46) (Table 3).

**Table 3:** Associations between Depression levels and different variables

			**Depression**				
**Item**	**Categories**	**N(%)**	**No depression (52)**	**Mild depression (38)**	**Moderate depression (21)**	**Severe depression (21)**	**χ2 (*P*-value)**
Gender	Male	45(38.5)	27(51.9)	11(28.9)	7(33.3)	0(0.0)	9.41(0.02)
	Female	72(61.5)	25(48.1)	27(71.1)	14(66.7)	6(100)	
Age	≤ 25 year	21(17.9)	8(15.4)	7(18.4)	5(23.8)	1(16.7)	10.5(0.57)
	26–35 year	74(63.2)	32(61.5)	21(55.3)	16(76.2)	5(83.3)	
	36–45 year	18(15.4)	10(19.2)	8(21.1)	0(0.0)	0(0.0)	
	45–55 year	3(2.6)	1(1.9)	2(5.3)	0(0.0)	0(0.0)	
	56–65 year	1(0.9)	1(1.9)	0(0.0)	0(0.0)	0(0.0)	
Marital Status	Single	55(47.0)	21(40.4)	15(39.5)	16(76.2)	3(50.0)	10.31(0.11)
	Married	56(47.9)	29(55.8)	20(52.6)	4(19.0)	3(50.0)	
	Divorced	6(5.1)	2(3.8)	3(7.9)	1(4.8)	0(0.0)	
Do you have children	Yes	39(33.3)	20(38.5)	17(44.7)	2(9.5)	0(0.0)	11.19(0.01)
	No	78(66.7)	32(61.5)	21(55.3)	19(90.5)	6(100)	
You live with your family	Yes	95(81.2)	46(88.5)	29(76.3)	16(76.2)	4(66.7)	3.56(0.31)
	No	22(18.8)	6(11.5)	9(23.7)	5(23.8)	2(33.3)	
Do you have a child between the age (0 to 6)	Yes	33(28.2)	18(34.6)	13(34.2)	2(9.5)	0(0.0)	7.70(0.05)
	No	84(71.8)	34(65.4)	25(65.8)	19(90.5)	6(100)	
Do you live with a family member aged ≥ 65	Yes	43(36.8)	19(36.5)	17(44.7)	5(23.8)	2(33.3)	2.58(0.46)
	No	74(63.2)	33(63.5)	21(55.3)	16(76.2)	4(66.7)	
Medical problem	Yes	16(13.7)	8(15.4)	2(5.3)	4(19.0)	2(33.3)	4.88(0.18)
	No	101(86.3)	44(84.6)	36(94.7)	17(81.0)	4(66.7)	
Current working region	Central region	68(58.1)	34(65.4)	19(50.0)	11(52.4)	4(66.7)	19.16(0.08)
	East region	11(9.4)	5(9.6)	3(7.9)	2(9.5)	1(16.7)	
	West region	24(20.5)	9(17.3)	13(34.2)	1(4.8)	1(16.7)	
	South region	7(6.0)	3(5.8)	1(2.6)	3(14.3)	0(0.0)	
	North region	7(6.0)	1(1.9)	2(5.3)	4(19.0)	0(0.0)	
what is your current practice setting	General hospital	81(69.2)	32(61.5)	32(84.2)	12(57.1)	5(83.3)	9.35(0.15)
	Private clinic	28(23.9)	14(26.9)	6(15.8)	7(33.3)	1(16.7)	
	Others	8(6.8)	6(11.5)	0(0.0)	2(9.5)	0(0.0)	
Years of clinical experience	< 2 year	24(20.5)	10(19.2)	7(18.4)	6(28.6)	1(16.7)	11.65(0.23)
	2 to 5 year	47(40.2)	17(32.7)	15(39.5)	12(57.1)	3(50.0)	
	5–10 year	22(18.8)	11(21.2)	6(15.8)	3(14.3)	2(33.3)	
	≥ 10 year	24(20.5)	14(26.9)	10(26.3)	0(0.0)	0(0.0)	
Isolation experience	Yes	37(31.6)	13(25.0)	12(31.6)	10(47.6)	2(33.3)	3.54(0.31)
	No	80(68.4)	39(75.0)	26(68.4)	11(52.4)	4(66.7)	
Had any of your family members been infected with COVID-19	Yes	51(43.6)	23(44.2)	14(36.8)	12(57.1)	2(33.3)	2.53(0.46)
	No	66(56.4)	29(55.8)	24(63.2)	9(42.9)	4(66.7)	
working in-person care (in clinic) as a physical therapist during the pandemic?	Yes	80(68.4)	36(69.2)	26(68.4)	13(61.9)	5(83.3)	1.04(0.79)
	No	37(31.6)	16(30.8)	12(31.6)	8(38.1)	1(16.7)	
Taking care of COVID-19 patients	Yes	26(22.2)	10(19.2)	10(26.3)	6(28.6)	0(0.0)	2.84(0.41)
	No	91(77.8)	42(80.8)	28(73.7)	15(71.4)	6(100)	

The prevalence of overall anxiety levels was higher; mild (OR = 2.09; *P* = 0.08), moderate (OR = 2.26; *P* = 0.15), and severe levels six times high (OR = 6.28; *P* = 0.1) in females compared to male physical therapists. Divorced participants had higher (OR = mild- 3.67 time, moderate- 3.14 time, and severe- 2.14 time) anxiety levels compared to the married participants. A severe level of anxiety (OR = 3.2; *P* = 0.3) was found in those physical therapists with no children. Physical therapists living with their families had less anxiety than family members not living together (OR = mild-2, moderate-2.7, and severe-3.04) (Table 4).

**Table 4:** Binary logistic regression analysis for risk factor associated with Anxiety

Item	Categories	No anxiety (%)	Mild anxiety (%)	OR (95% CI)	*P*-value	Moderate anxiety (%)	OR (95% CI)	*P*-value	Severe anxiety (%)	OR (95% CI)	*P*-value
Gender	Male	22(48.9)	16(35.6)	|		6(13.3)	|		1(2.2)	|	
	Female	21(29.2)	32(44.4)	2.09(0.89–4.8)	0.08	13(18.1)	2.26(0.7–7.0)	0.15	6(8.3)	6.28(0.69–56.7)	0.1
Marital Status	Single	20(36.4)	20(36.4)	0.91(0.39–2.1)	0.84	11(20.0)	1.72(0.56–5.3)	0.34	4(7.3)	1.46(0.29–7.3)	0.64
	Married	22(39.3)	24(42.9)	|		7(12.5)	|		3(5.4)	|	
	Divorced	1(16.7)	4(66.7)	3.67(0.38–35.3)	0.26	1(16.7)	3.14(0.17–57)	0.43	0(0)	2.14(0.07–63.7)	0.65
Do you have children	Yes	15(38.5)	18(46.2)	|		5(12.8)	|		1(2.6)	|	
	No	28(35.9)	30(38.5)	0.89(0.37–2.10)	0.79	14(17.9)	1.5(0.45–4.9)	0.5	6(7.7)	3.2(0.35–29.2)	0.3
Live with family	Yes	38(40.0)	38(40.0)	|		14(14.7)	|		5(5.3)	|	
	No	5(22.7)	10(45.5)	2.0(0.62–6.4)	0.24	5(22.7)	2.7(0.68–10.8)	0.15	2(9.1)	3.04(0.46–20.0)	0.24
Any child between the age (0–6)	Yes	12(36.4)	15(45.5)	|		5(15.2)	|		1(3.0)	|	
	No	31(36.9)	33(39.3)	0.85(0.34–2.1)	0.72	14(16.7)	1.08(0.32–3.6)	0.89	6(7.1)	2.32(0.25–21.3)	0.45
Any family member aged over 65	Yes	16(37.2)	18(41.9)	|		7(16.3)	|		2(4.7)	|	
	No	27(36.5)	30(40.5)	0.98(0.42–2.31)	0.97	12(16.2)	1.0(0.33–3.10)	0.97	5(6.8)	1.48(0.25–8.54)	0.66
Any Medical Problem	Yes	7(43.8)	5(31.3)	0.59(0.17–2.04)	0.41	2(12.5)	0.60(0.11–3.2)	0.56	2(12.5)	2.05(0.33–12.8)	0.43
	No	36(35.6)	43(42.6)	|		17(16.8)	|		5(5.0)	|	
Current practice setting	General hospital	29(35.8)	35(43.2)	|		14(17.3)	|		3(3.7)	|	
	Private hospital	8(28.6)	13(46.4)	1.34(0.49–3.69)	0.56	5(17.9)	1.29(0.35–4.6)	0.69	2(7.1)	2.41(0.34–17.0)	0.37
	Others	6(75.0)	0(0.0)	0		0(0.0)	0		2(25.0)	3.2(0.43–23.6)	0.25
Years of experience	< 2	8(33.3)	8(33.3)	1.50(0.36–6.23)	0.57	5(20.8)	1.12(0.23–5.3)	0.88	3(12.5)	1.68(0.22–12.8)	0.61
	2 to 5 year	15(31.9)	24(51.1)	2.40(0.71–8.1)	0.15	6(12.8)	0.72(0.16–3.0)	0.65	2(4.3)	0.60(0.07–5.0)	0.63
	5 to 10 year	9(40.9)	6(27.3)	|		5(22.7)	|		2(9.1)	|	
	> 10 year	11(45.8)	10(41.7)	1.36(0.35–5.21)	0.65	3(12.5)	0.49(0.09–2.6)	0.4	0(0.0)	0.16(0.007–3.8)	0.26
Isolation experience	Yes	9(24.3)	18(48.6)	2.26(0.88–5.7)	0.08	7(18.9)	2.20(0.67–7.2)	0.19	3(8.1)	2.83(0.53–15.0)	0.22
	No	34(42.5)	30(37.5)	|		12(15.0)	|		4(5.0)	|	
Any family members had COVID-19	Yes	22(43.1)	17(33.3)	|		10(19.6)	|		2(3.9)	|	
	No	21(31.8)	31(47.0)	1.9(0.82–4.4)	0.13	9(13.6)	0.94(0.31–2.7)	0.91	5(7.6)	2.61(0.45–15.0)	0.27
Have you working person care during COVID-19	Yes	27(33.8)	34(42.5)	1.43(0.59–3.4)	0.41	14(17.5)	1.65(0.50–5.4)	0.4	5(6.3)	1.48(0.25–8.5)	0.67
	No	16(43.2)	14(37.8)	|		5(13.5)	|		2(5.4)	|	
Have you currently care COVID patients	Yes	11(42.3)	11(42.3)	|		4(15.4)	|		0(0.0)	|	
	No	32(35.2)	37(40.7)	1.15(0.44–3.02)	0.76	15(16.5)	1.28(0.35–4.7)	0.7	7(7.7)	0	

Less anxiety was found in the current study for the participants with a family member aged 65 years or older. The prevalence of severe anxiety levels (OR = 2.05; *P* = 0.43) was higher among those with a medical problem. Those with isolation experience had more anxiety (OR = mild 2.26, moderate 2.20, and severe 2.83 times) than those with no isolation experience (Table 4).

The prevalence of exposed depression in the current study found that females had 14 times more severe depression levels than male physical therapists (OR = 14; *P* = 0.07). Also, the divorced participants had higher (OR = mild-2.1 times, moderate-2.6 times, and severe-1.22 times) depression levels than the married participants. There was significantly 5.5 times more moderate level depression among single participants (OR = 5.5, *P* = 0.006). The prevalence of depression was higher (OR = moderate-5.9, and severe-8.2) among those physical therapists who had no children. Those who lived with their family had less depression (OR = mild-2.3, moderate-2.3, and severe-3.8) (Table 5).

**Table 5:** Binary logistic regression analysis for risk factor associated with Depression

Item	Categories	No depression (%)	Mild depression (%)	OR (95% CI)	*P*-value	Moderate depression (%)	OR (95% CI)	*P*-value	Severe depression (%)	OR (95% CI)	*P*-value
Gender	Male	27(60)	11(24.4)	|		7(15.6)	|		0(0.0)	|	
	Female	25(34.7)	27(37.5)	2.65(1.09–6.4)	0.03	14(19.4)	2.16(0.74–6.2)	0.15	6(8.3)	14(0.75–261)	0.07
Marital status	Single	21(38.2)	15(27.3)	1.03(0.43–2.5)	0.93	16(29.1)	5.5(1.6–18.9)	0.006	3(5.5)	1.38(0.25–7.5)	0.7
	Married	29(51.8)	20(35.7)	|		4(7.1)	|		3(5.4)	|	
	Divorced	2(33.3)	3(50.0)	2.1(0.31–14.1		1(16.7)	2.6(0.18–36.3)	0.47	0(0.0)	1.22(0.04–31.3)	0.9
Do you have children	Yes	20(51.3)	17(43.6)	|		2(5.1)	|		0(0.0)	|	
	No	32(41.0)	21(26.9)	0.77(0.33–1.8)	0.55	19(24.4)	5.9(1.2–28.3)	0.02	6(7.7)	8.2(0.4–153.4)	0.15
Live with family	Yes	46(48.4)	29(30.5)	|		16(16.8)	|		4(4.2)	|	
	No	6(27.3)	9(40.9)	2.37(0.76–7.3)	0.13	5(22.7)	2.39(0.64–8.9)	0.19	2(9.1)	3.83(0.57–25.5)	0.16
Any child between the age (0–6)	Yes	18(54.5)	13(39.4)	|		2(6.1)	|		0(0.0)	|	
	No	34(40.5)	25(29.8)	1.01(0.42–2.4)	0.96	19(22.6)	5.02(1.05–24)	0.04	6(7.1)	6.9(0.37–130)	0.19
Any family member aged over 65	Yes	19(44.2)	17(39.5)	|		5(11.6)	|		2(4.7)	|	
	No	33(44.6)	21(28.4)	0.71(0.30–1.6)	0.43	16(21.6)	1.84(0.58–5.8)	0.29	4(5.4)	1.15(0.19–6.8)	0.87
Any Medical Problem	Yes	8(50.0)	2(12.5)	0.30(0.06–1.5)	0.14	4(25.0)	1.29(0.34–4.8)	0.7	2(12.5)	2.75(0.42–17.6)	0.28
	No	44(43.6)	36(35.6)	|		17(16.8)	|		4(4.0)	|	
Current practice setting	General hospital	32(39.5)	32(39.5)	2.34(0.79–6.8)	0.12	12(14.8)	0.75(0.24–2.3)	0.61	5(6.2)	2.18(0.23–20.4)	0.49
	Private hospital	14(50.0)	6(21.4)	|		7(25.0)	|		1(3.6)	|	
	Others	6(75.0)	0(0.0)	0		2(25.0)	0.66(0.10–4.1)	0.67	0(0.0)	0	
Years of experience	< 2	10(41.7)	7(29.2)	1.28(0.32–5.1)	0.72	6(25.0)	2.20(0.43–11.2)	0.34	1(4.2)	0.55(0.04–7.0)	0.64
	2 to 5 year	17(36.2)	15(31.9)	1.61(0.48–5.4)	0.43	12(25.5)	2.58(0.59–11.3)	0.2	3(6.4)	0.97(0.13–6.7)	0.97
	5 to 10 year	11(50.0)	6(27.3)	|		3(13.6)	|		2(9.1)	|	
	> 10 year	14(58.3)	10(41.7)	1.30(0.36–4.7)	0.68	0(0.0)	0		0(0.0)	0	
Isolation experience	Yes	13(35.1)	12(32.4)	1.26(0.51–3.0)	0.6	10(27.0)	1.90(0.72–5.1)	0.19	2(5.4)	1.34(0.24–7.3)	0.74
	No	52(44.4)	38(32.5)	|		21(17.9)	|		6(5.1)	|	
Any family members had COVID-19	Yes	23(45.1)	14(27.5)	0.73(0.31–1.7)	0.48	12(23.5)	1.68(0.60–4.6)	0.31	2(3.9)	0.63(0.10–3.7)	0.61
	No	29(43.9)	24(36.4)	|		9(13.6)	|		4(6.1)	|	
Have you working person care during COVID-19	Yes	36(45.0)	26(32.5)	0.96(0.3–2.37)	0.93	13(16.3)	0.72(0.25–2.0)	0.54	5(6.3)	2.2(0.23–20.5)	0.48
	No	16(43.2)	12(32.4)	|		8(21.6)	|		1(2.7)	|	
Have you currently care COVID patients	Yes	10(38.5)	10(38.5)	0.67(0.24–1.8)	0.42	6(23.1)	1.68(0.52–5.4)	0.38	0(0.0)	|	
	No	42(46.2)	28(30.8)	|		15(16.5)	|		6(6.6)	0	0.43

The prevalence of severe levels of depression was three times high (OR = 2.75; *P* = 0.28) among participants with a medical problem. Those with more physical therapy experience had less depression, less than two years experience (OR = 2.20; *P* = 0.34), and 2 to 5 years of experience (OR = 2.58; *P* = 0.2) had a moderate level of depression. The physical therapists with isolation experience had more depression (OR = mild-1.26, moderate-1.90, and severe-1.34) than those with no isolation experience. Those physical therapists who were treating COVID-19 patients had a moderate level of depression (OR = 1.68; *P* = 0.38) (Table 5).

## Discussion

This study aimed to investigate the psychological status and mental health burden among Saudi physical therapists during the COVID-19 outbreak. Study results revealed a high prevalence of anxiety and depression among participants (63 and 55.5%, respectively). The findings are higher than a recent national study as they found a 28.7 and 25.5% prevalence of depression and anxiety among the general population during the COVID-19 outbreak based on PHQ-9 and GAD-7 surveys [23]. In another national study where only 2.2% of physical therapists among HCWs reported anxiety symptoms during the pandemic [24]. The low prevalence of anxiety among physical therapists compared with the present study is probably because they conducted their study on mixed health workers' specialties, the proportion of physical therapists was very low in number.

Furthermore, it was found that those physical therapists with any family member over the age of 65 have anxiety levels that are more than 1.48% higher (severe), moderate and severe depression levels that are 1.84 and 1.15% higher, respectively. Compared with a recent study that reported a prevalence of mental health symptoms among Korean physical therapists, reported 32.3 and 18.5% symptoms of anxiety and depression if they have old person in their family [1]. It may be particularly important to monitor the mental health of young and economically vulnerable individuals in the future. Moreover, they indicate that the general public's mental health may also be affected by the economic ramifications of COVID-19 during the pandemic as well as its direct health consequences. Anxiety, depression, insomnia, psychological distress, and other mental health issues have been investigated in recent epidemiological studies conducted during the outbreak [25]. This prevalence has increased in a recent study which reported a higher prevalence of anxiety and depression by 47.6 and 44% among South Korean Physical Therapists [26]. This suggested an interesting view that prolonged exposure to the virus in the medical field as the pandemic persisted and anxiety and depression levels could have evolved in physical therapists before and during the crisis. Also, one would predict that the anxiety and depression levels would drop in number, but on the other hand, there may be an increase in reporting of post-traumatic stress symptoms [22].

Many were associated with high anxiety and depression levels; younger age, female gender, single or divorced status, living with a chronic disease, a previous history of isolation, and not living with their family. These findings are consistent with other studies looking at the impact of COVID-19 on the mental health of HCWs [24, 27].

A higher rate of anxiety and depression was found among the younger generation. This is supported by other national studies where the tendency to have psychological distress and adverse mental health was higher among younger adults [23, 28]. In addition, prior studies were consistent with the current results indicating that during the COVID-19 pandemic, the prevalence rate of depression was higher for younger physical therapists [1, 26]. Several explanations were offered, including access to a vast amount of information via social media and the stronger effect of lockdown on younger people [29]. It is also possible that older physical therapists are more experienced and better equipped both professionally and psychologically to deal with the stress of the pandemic than younger physical therapists since less experience was a risk factor for stress among HCWs during the COVID-19 pandemic [24, 30].

The female gender was associated with higher depression levels consisting of similar corroborating studies [24, 31, 32]. The significantly increased depression levels among female physical therapists can reflect the high proportion of females in this sample to male participants. Also, it is well established in previous studies that females are more likely to report symptoms of common mental health problems, including depression and anxiety [33, 34].

Other demographic factors may contribute to anxiety and depression, such as unmarried individuals, family members who do not live together, and previous isolation experiences. A convergence result was found on the increased risk of post-traumatic stress symptoms in medical staff who are unmarried, divorced, or widowed [35]. On the other hand, conflicting findings were reported by Alenazi et al., 2020 and Al Ammari et al., 2020, that living with family members was predicted to increase anxiety [24, 27].

This is probably because those physical therapists might receive less care and communication from their partners and may experience less family support. Other studies highlighted that showed an increased risk of depression, anxiety, stress, and post-traumatic stress symptoms in HCWs who lacked social and emotional support from their family [26, 36, 37].

Furthermore, in this study, anxiety and depression were not associated with having children. These results are conflicting with findings reported by Al Ammari et al., 2020 and Yang et al., 2020, where having children was a risk factor for developing depression and anxiety. Our findings can be explained by a recent study that reported that children infected with COVID-19 were less likely to have severe symptoms or serious diseases [38]. This could have affected the perception of families with young children to be less anxious and concerned about the transmission of COVID-19. Interestingly, although most of the participants were working in-person care, treating COVID-19 patients did not associate with anxiety and depression, conflicting with the results of other studies with HCWs [14, 24]. This controversial result is probably because the small number of physical therapists took care of COVID-19 patients in the present study. It could be insufficient to show a statistical difference since the number of physical therapists treating COVID-19 patients was low.

The present study is the first study investigating anxiety and depression among Saudi physical therapists during the COVID-19 pandemic from different regions in Saudi Arabia and from various fields to be representative. Moreover, the timing of the study was appropriate because a large number of cases of COVID-19 were reported in the country at the time. On the other hand, the study has a few limitations. First, the sample size was small, whichmight be attributed to the narrow inclusion criteria where physical therapists with previous diagnoses of psychiatric disorders and a history of confirmed infection of COVID-19 were excluded and did not have the chance to participate. However, we think the high response rate (89.3%) compensated for the low sample size and achieved the desired power. Second, the study did not employ a longitudinal follow-up, considering the probability of an increased number of confirmed COVID-19 and post-traumatic stress symptoms in physical therapists facing the current COVID-19 pandemic. Third, the study design did not include cause analysis for psychological strains, such as work-related stress. Future research is needed to address the above limitations, including conducting a prospective study on the same physical therapists after a period to handle the long-term courses of anxiety and depression.

## Conclusion

The pandemic of COVID-19 has caused a serious emerging challenge for the general population and health professionals worldwide. Considering a significant percentage of physical therapists reported symptoms of anxiety and depression, the mental health of physical therapists should be constantly and cautiously monitored. Based on the findings of this study, it is also necessary to provide physical therapists at high risk, such as females, younger aged and unmarried individuals, more psychological support and communication to alleviate their overall psychological well-being. Physical therapists should be offered financial incentives to reduce their workload based on the results of this research. Furthermore, physical therapists may obtain urgent appointments (if and when needed) without prior booking in collaboration with psychiatry and psychology units.

## Data Availability

All datasets used and/or analyzed in this study are available upon request from the corresponding author.

## Abbreviations

GAD: Generalized anxiety disorder scale
PHQ: Patients were asked to complete the patient health questionnaire
SARS-CoV: Severe acute respiratory syndrome coronavirus
COVID-19: Coronavirus disease-19
HCW: Health-care workers
IRB: Institutional review board
KSU: King Saud University
SPSS: Statistical package for the social sciences
UK: United Kingdom
USA: United States of America
UAE: United Arab Emirates
